# Supplementary material for: TiO2 Nanotube Layers Decorated with Al2O3/MoS2/Al2O3 as Anode for Li-ion Microbatteries with Enhanced Cycling Stability
Source: Nanomaterials (Basel). 2020 May 17;10(5):953. doi: 10.3390/nano10050953 (PMC7279526; doi:10.3390/nano10050953)
Supplement: Supplementary file 1 [file nanomaterials-10-00953-s001.pdf]

nanomaterials

Supplementary Materials:

TiO_2 Nanotube Layers Decorated with $Al_2O_3/MoS_2/Al_2O_3$ as Anode for Li-ion Microbatteries with Enhanced Cycling Stability

Alexander Teklit Tesfaye[1], Hanna Sopha[2,3], Angela Ayobi[1], Raul Zazpe[2,3], Jhonatan Rodriguez-Pereira[2], Jan Michalicka[3], Ludek Hromadko[2,3], Siowwoon Ng[3], Zdenek Spotz[3], Jan Prikryl[2], Jan M. Macak[2,3], and Thierry Djenizian[1,4*]

[1] Mines Saint-Etienne, Center of Microelectronics in Provence, Flexible Electronics Department, 13541 Gardanne, France ; alexanderteklit@gmail.com, angieayobi@gmail.com
[2] Center of Materials and Nanotechnologies, Faculty of Chemical Technology, University of Pardubice, Nam. Cs. Legii 565, 53002 Pardubice, Czech Republic; HannaIngrid.Sopha@upce.cz, Raul.Zazpe@upce.cz, Jhonatan.RodriguezPereira@upce.cz, Ludek.Hromadko@upce.cz, Jan.Prikryl@upce.cz, Jan.Macak@upce.cz
[3] Central European Institute of Technology, Brno University of Technology, Purkyňova 123, 612 00 Brno, Czech Republic; jan.michalicka@ceitec.vutbr.cz SiowWoon.Ng@ceitec.vutbr.c,Zdenek.Spotz@ceitec.vutbr.cz
[4] Al-Farabi Kazakh National University, Center of Physical-Chemical Methods of Research and Analysis, Tole bi str., 96A. Almaty, Kazakhstan.
* Correspondence: thierry.djenizian@emse.fr; Tel.: +33-623 75 03 44

Received: date; Accepted: date; Published: date

Keywords: TiO_2 nanotube; MoS_2; Al_2O_3; Atomic Layer Deposition; Li-ion microbatteries

---

Calculation to determine the porosity of TNTs

The porosity calculation is based on the amount of TiO_2 nanotubes per cm^{-2} on obtained SEM images. In average, there are 46.83 nanotubes per μm^2. The nanotube diameters were measured using our proprietary software "nanomeasure". In average, the inner and outer diameter of the nanotubes was 120.95 nm and 150.54 nm, respectively. Area of the TNTs was calculated by assuming the nanotubes have a shape of a circle at the top.

$$Area_{TNT} = \pi*(R^2 - r^2)$$

Where,

$Area_{TNT}$ = Area of a single TiO_2 nanotubes
R and r = outer and inner diameter of TiO_2 nanotubes

$$A_{TNT} = 6309.41\ nm^2$$

So, the area occupied by TNTs on planar area of 1 cm^2 is,

$$Area_{TNT/cm^2} = \text{Number of nanotubes per } cm^{-2} * Area_{TNT}$$
$$= 0.295$$

Therefore, the free area is 70.5 %.

Figure S1 shows the EDX spectrum of $Al_2O_3/MoS_2/Al_2O_3$-TNTs before and after 100 cycles at The presence of Mo, Al, S, Ti, and O peaks confirms the $Al_2O_3/MoS_2/Al_2O_3$ surface coating and the underlying self-supporting TNTs. The additional C and Cu peaks are attributed to the membrane

and the TEM sample grid. Fe, Co, and Zr peaks are from the TEM column and Si is ascribed to the SDD EDX detectors.

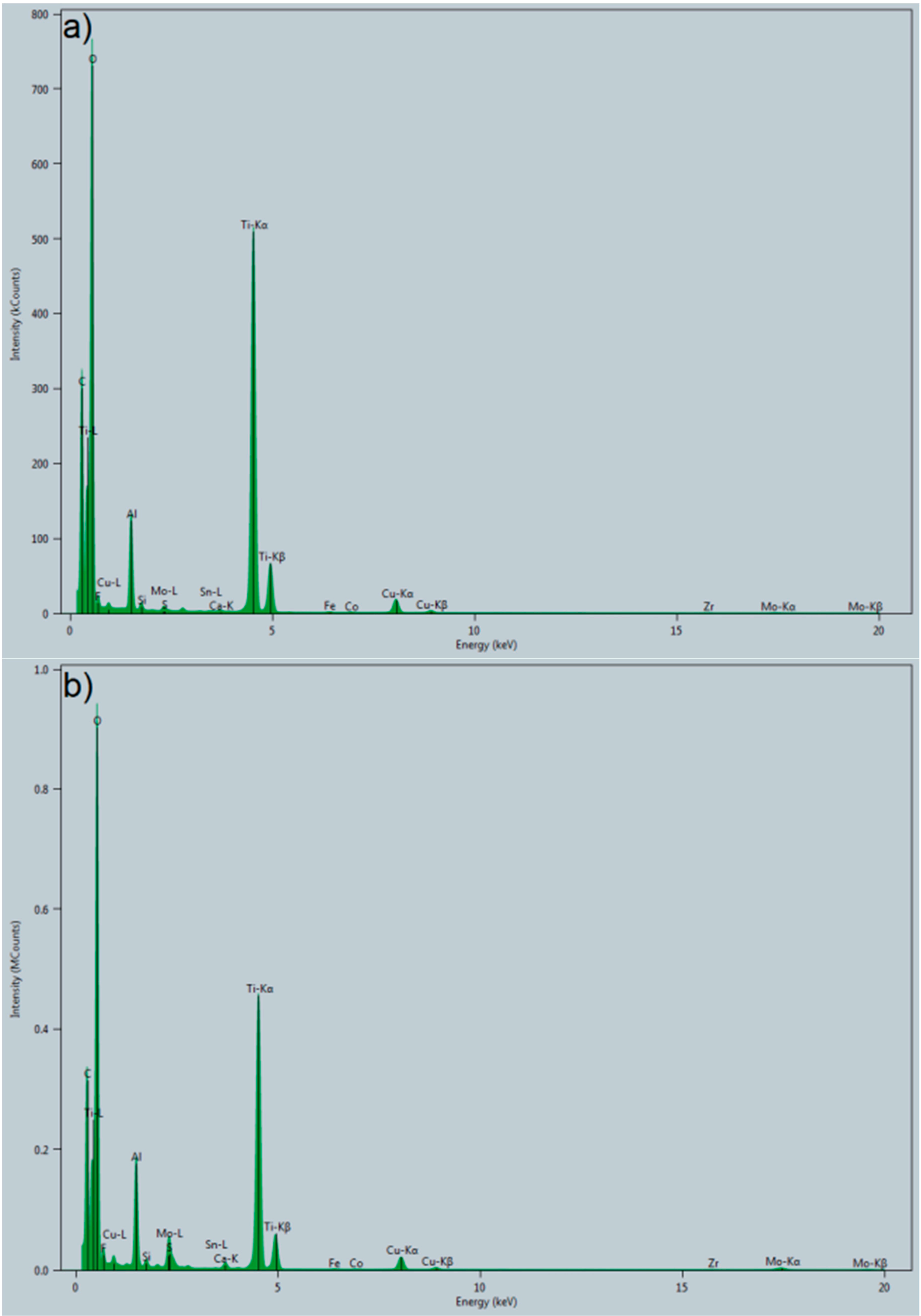

Figure S1. EDX spectrum of $Al_2O_3/MoS_2/Al_2O_3$-TNTs (a) before and (b) after 100 galvanostatic cycles.
